# Long term dual antiretroviral therapy: A real life retrospective countrywide Israeli study

**DOI:** 10.1371/journal.pone.0259271

**Published:** 2021-10-29

**Authors:** Daniel David, Eynat Kedem, Dan Turner, Itzchak Levy, Daniel G. Elbirt, Eduardo Shahar, Valery Istumin, Orna Mor, Michal Chowers, Hila Elinav

**Affiliations:** 1 The Hebrew University Hadassah Medical School, Jerusalem, Israel; 2 Institute of Allergy, Immunology and AIDS Rambam Medical Center, Haifa, Israel; 3 Crusaid Kobler AIDS Center, Tel-Aviv Sourasky Medical Center, Tel-Aviv, Israel; 4 Sackler Faculty of Medicine, Tel Aviv University, Tel Aviv, Israel; 5 Infectious Disease Unit, Chaim Sheba Medical Center, Ramat Gan, Israel; 6 The Allergy, Clinical Immunology and AIDS Unit, Kaplan Medical Center, Rehovot, Israel; 7 HIV Service, Internal Medicine C Department, Hillel Yaffe Medical Center, Hadera, Israel; 8 Central Virology Laboratory, Ministry of Health, Public Health Services, The Chaim Sheba Medical Center, Tel Hashomer, Ramat-Gan, Israel; 9 Infectious Diseases Unit, Meir Medical Center, Kfar Saba, Israel; 10 Department of Clinical Microbiology and Infectious Diseases, Hadassah AIDS Center, Hadassah Hebrew University Medical Center, Jerusalem, Israel; Consejo Superior de Investigaciones Cientificas, SPAIN

## Abstract

**Aim:**

Combined antiretroviral treatment (cART) traditionally consists of three antiretroviral medications, while two-drug regimens (2DR), historically used infrequently, recently been suggested to be non-inferior to three-drug regimens, is emerging as a potential treatment option and is currently a recommended option for treatment initiation in many guidelines.

**Purpose:**

Characterize the indications and clinical efficacy of 2DR use at a real-life setting in a nation-wide survey.

**Methods:**

A cross-sectional survey of Israeli patients treated by 2DR until July 2019, included demographic, immunologic, virologic, genotypic and biochemical/metabolic parameters at diagnosis, ART initiation, 2DR initiation and following 24, 48, 96 and 144 weeks of 2DR treatment.

**Results:**

176 patients were included in the study. In contrast to historical data implicating ART resistance and adverse effects as the major reasons leading to 2DR switching, treatment simplification was the main reason leading to 2DR treatment in 2019. 2DR that included INSTI and PI were more commonly used in cases of drug resistance, while a combination of INSTI and NNRTI was used in all other 2DR indications. A switch to 2DR induced a mean CD4 T cell increase from 599 cells/μl at treatment initiation to 680 cells/μl at 96 weeks of treatment p<0.001 and viral suppression improvement from 73.9% at initiation to 87.0% at 48 weeks of treatment (p = 0.004). PI and INSTI 2DR was inferior in suppressing viral levels compared to other 2DRs but used for subset of more complex patients.

**Conclusions:**

2DR in a large-scale real-life nation-wide survey proved to be safe and effective. Most 2DRs, other than PI and INSTI, were similarly effective in suppressing HIV viremia and in elevating CD4 T cell counts.

## Introduction

Combined antiretroviral treatment (cART) evolved from a single drug therapy [1] to a triple drugs combination that inhibit HIV replication by distinct mechanisms of activity and enabled to achieve long durable viral suppression [2, 3]. Consequently, ART comprised of 2 NRTIs plus non nucleotide analog reverse transcriptase inhibitor (NNRTI), protease inhibitor (PI) or integrase inhibitor (INSTI), became the common practice in the management of HIV patients [4]. cART has been proven to suppress viral replication, improve clinical outcomes and decrease mortality among HIV infected patients, thereby converting HIV from a terminal disease to a chronic and manageable disorder [5, 6]. With the increase in life expectancy and long term medication exposure, multiple adverse effects, including cardiovascular, renal, neurological, and bone manifestations developed in chronically treated people living with HIV [7]. In some cases of patients suffering of severe adverse effects and multiple drugs viral resistance, unavailability of better options dictated the use of dual effective antiretroviral therapy. The encouraging results obtained with dual therapy in these selected patients and the desire to minimize drug toxicities were the rational for multiple clinical trials studying the effect of dual and monotherapy in HIV [8], with controversial results noted [9–12]. The improved efficacy of INSTIs recently enabled to reassess the dual ART option, with an aim to maintain adequate virologic suppression with reduced incidence of adverse effects in selected cases [13–15]. Moreover, the encouraging results of the SWORD-1 and SWORD-2 trials [16, 17], motivated some clinicians to switch patients to dual therapy even in cases of clinically stable disease, in order to simplify ART and avoid future adverse effects.

A total of 7970 people living with HIV in Israel, are currently followed and treated in eight HIV centers. The aim of this study was to evaluate the real-life usage of two drug regimens (2DR) in Israel, to characterize the different regimens used in different HIV centers, the rationale leading to switching to 2DR, and the clinical outcomes of this practice.

## Methods

Patient information was retrieved from the local databases of seven Israeli HIV centers between July 2019 to August 2019. Included were all the HIV patients who were treated by two drug regimens (2DR) at the time point of data collection in these centers (up to 31.7.2019, n = 176). No exclusion criteria were implemented to reflect the real-life implications of dual therapy. The data collected included demographic, immunologic, virologic, resistance profile and biochemical/metabolic parameters at diagnosis, ART initiation, 2DR initiation and after 24, 48, 96 and 144 weeks of 2DR treatment±8 weeks. Immunologic and virologic data included CD4 count (cells/μl), CD8 count (cells/μl), HIV viral load (VL, copies/ml), Hepatitis B virus (HBV) and Hepatitis C virus (HCV) serology. The biochemical/metabolic parameters included renal function (creatinine levels, mg/dl), liver aminotransferase activity (AST & ALT levels, IU/ml), lipid profile, including triglycerides (TG, mg/dl), total cholesterol (mg/dl), low density lipoprotein (LDL, mg/dl), high density lipoprotein levels (HDL, mg/dl), and glucose levels (mg/dl). We documented the indication/s for 2DR initiation, the type of ART used before and after the switch to 2DR, the number of regimens that the patient has been treated with until 2DR. Resistance mutations documented at diagnosis, during follow up to 2DR switching and after 2DR initiation. Drug-resistance mutations were identified using the Stanford University HIV Drug-Resistance Database [18]. Changing treatment from two pills to a similar single tablet regimen (STR) was not considered as a switch to a new regimen. Undetectable viral load was defined as below detection limit according to the kit used by the HIV virology lab. Patients that featured a documented follow-up period of less than 24 weeks from initiation of dual therapy to data collection, and have therefore undergone only a descriptive analysis, while not being included in the 2DR efficacy analysis. For the analysis of annual switching to 2DR, we included patients who were switched from 1.8.2019 to 31.12.2019. These patients were not included in any other analysis.

### Statistical analysis

To evaluate the clinical efficacy of 2DR therapy over time, repeated measures ANOVA model was applied for assessment of quantitative variables. Assessing trend over time for quantitative variables which were not normally distributed, was performed using the non-parametric Friedman test. Testing the change between two time points for quantitative variables, was performed using a paired t-test. Testing the change between two time points for categorical variables, was performed by applying the McNemar or the McNemar-Bowker test. Pearson’s Chi-square test and Fisher’s exact test were used for testing the association between two categorical variables. All statistical tests were two-tailed and a p-value of less than 0.05 was considered statistically significant. Statistical analysis was carried out using IBM SPSS Statistics version 24.0.

### Ethics

The study was approved by IRB committees of all participating HIV Centers.

Approval number of Hadassah Medical center IRB 0574-18-HMO, approval number of Rambam Medical Center IRB 0428-19-RMC, approval number of Kaplan Medical Center IRB 0036-10-KMC, approval number of Meir Medical Center 001-19-MMC, approval number of Sourasky Medical Center IRB 0741-18-TLV, approval number of Sheba Medical Center IRB 5838-19-SMC. No approval was required from Hillel Yaffe Medical Center as no patients were treated with 2DR at the study data collection. All committees granted a formal waiver of informed consents as data was retrospective and analyzed anonymously.

## Results

**Fig 1:**
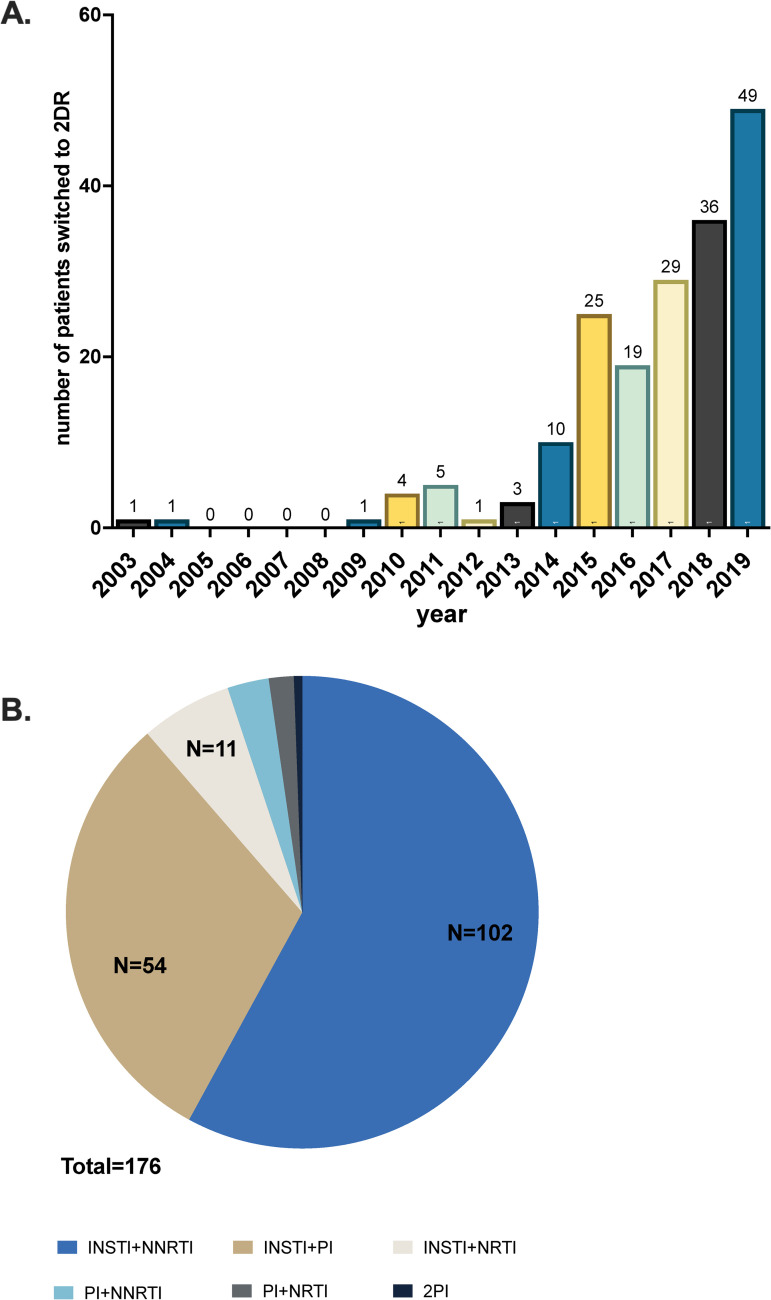
2DR initiation. A: Yearly number of patients that started/ switched to treatment with dual therapy. B: Dual regimens used by class. INSTI- integrase inhibitor, NNRTI- non nucleoside analog reverse transcriptase inhibitor, PI- protease inhibitor, NRTI- nucleoside analog reverse transcriptase inhibitor.

In reviewing all the medical records of patients living with HIV and followed by the participating 7 HIV centers, we identified 173 patients who were switched to dual therapy, and 3 patients who initiated 2 antiretroviral drug regimens as their primary ART regimen until 31.7.2019. In total, we were able to survey the data of 94% of 7970 people living with HIV in Israel, as one HIV center did not participate in the study. The rate of patients treated with dual therapy was comparable between the centers and ranged from 1.6% to 4%. Seventy-one precents of the patients were males and 40% were born in Israel, while Ethiopia was the main country of origin for immigrants. Patients were previously heavily treated for a mean of 8.57 years and a mean number of treatment switches totaled 2.08 as indicated in Table 1. Although the first switch to dual therapy was performed in 2003, further switching to 2DR was incidental until 2014 and a real escalation of the usage of 2DR was noted only in 2015 (Fig 1A). More than half of the patients (55.1%) were switched to dolutegravir (DTG) plus rilpivirine (RPV), with the second most common combination being dolutegravir (DTG) plus boosted darunavir (DRV) (18.2%). The rest of the regimens varied (Fig 1B).

**Table 1:** Basic characteristics of study participants.

**Category**		**Frequency (N)**		**Prevalence (%)**					
Gender M/F		125/51		71.0%/29%					
Country of birth	Israel	70		39.8					
	Africa	53		30.1					
	Europe	37		21					
	America	9		5.1					
	Asia	7		4.0					
HBV +/-/unk		2/167/8		1.1/94.4/4.5					
HCV +/-/unk		14/155/8		7.9/87.6/4.5					
**Category**	**N**	**Mean**	**Median**	**Std. Dev.**	**Range**				
Age at Diagnosis	171	37.02	35	12	1–69				
Age at beginning of ART	143	39.76	38	11.5	1–69				
CD4 at diagnosis cell/μ	104	360.46	331.5	280.6	3–1181				
Nadir CD4 cell/μ	120	219.5	179	186.2	3–775				
CD4 at treatment initiation cell/μ	119	287.5	259	230	3–1181				
Years of treatment before 2DR initiation	143	8.57	7.0	6.3	0–24				
Number of ART switches before 2DR	152	2.08	2	2.04	0–13				
Age at 2DR initiation	176	49.1	49.5	12.1	18–80				
CD4 at 2DR initiation	170	569.18	525.0	305.1	3–1536				
VL at 2DR treatment initiation copies/ml	169	23093	0	154201	0–1.5*10^7^				
major mutation N (%)									
ART	48 (27.3)								
NRTI	43 (24.4)								
NNRTI	26 (14.8)								
PI	18 (10.2)								
INSTI	4 (2.3)								
**Treatment before 2DR**	**N**	**Age at 2DR initiation** mean (SD)	**Years of ART before 2DR** mean (SD)	**# of previous switches** mean (SD)	**CD4 at 2DR initiation** mean (SD)	**VL at 2DR initiation** mean (SD)	**% VL detec**	**Reason for 2DR switch (1** ^**st**^ **+2** ^**nd**^ **) (%)**	**% Of patients with ART mutation**
2 NRTI + INSTI	84	49.18 (12.8)	6.7 (5.6)	1.64 (1.64)	607.0 (312)	17341 (123367)	21.0	renal (27.4) simp (27.4)	19
2 NRTI + PI	27	46.81 (9.845)	9.2 (5.7)	1.75 (1.15)	423.9 (269.9	23754 (71110)	40.7	resis (51.9) renal (44.4)	48.1
2 NRTI +NNRTI	26	49.27 (12.7)	10.9 (5.9)	2.13 (2.6)	561.6 (250.6)	63736 (325100)	21.7	renal (23.1 osteo (19.2)	15
1/2 NRTI + INSTI + PI	11	48.2 (11.3)	8.6 (5.78)	3.33 (2.4)	543.2 (208.7)	9743 (32256)	27.3	resis (27.3) renal (27.3) simp (27.3)	54.6
NNRTI + INSTI + PI	9	51.5 (7.4)	15.2 (5.06)	4.13 (2.6)	684.3 (438.5)	0 (0)	0	resis (44.4) simp (22)	66.7
1/2 NRTI+ NNRTI + INSTI	4	56.5 (11.7)	17.25 (3.18)	4.0 (2.8)	702.3 (413.5)	0 (0)	0	simp (75) cardi (25) lipid (25)	0
1/2 NRTI +NNRTI +PI	3	65.3 (9.2)	19.0 (1.4)	4.5 (3.5)	690.7 (190.3)	0 (0)	0	simp (33) neuro (33.5) osteo (33.3)	33
NNRTI + INSTI + CCR5-in	1	59	22.5	5	525	0	0	simp	0
NNRTI + PI + CCR5-in	1	51	5.5	3	1159	0	0	simp	0
Unknown	7	45.1 (13.6)	5.2 (4.1)	1.8 (2.0)	523.7 (260.9)	573.3 (1096)	66.6	-	28.6
Naive	3	39.6	0	0	328	55125			

Basic characteristics of patients including in the study, including all the patients, and stratified according to treatment regimen before 2DR.

INSTI- integrase inhibitor, NNRTI- non nucleoside analog reverse transcriptase inhibitor, PI- protease inhibitor, NRTI- nucleoside analog reverse transcriptase inhibitor. CCR5 in- CCR5 inhibitor, unk- unknown. #- number, % VL Detec- % of patients with detectable VL at switch, resis- resistance, osteo- osteoporosis, simp- simplification, cardi- cardiovascular risk or disease, lipid- hyperlipidemia, neuro- neurological.

**Fig 2:**
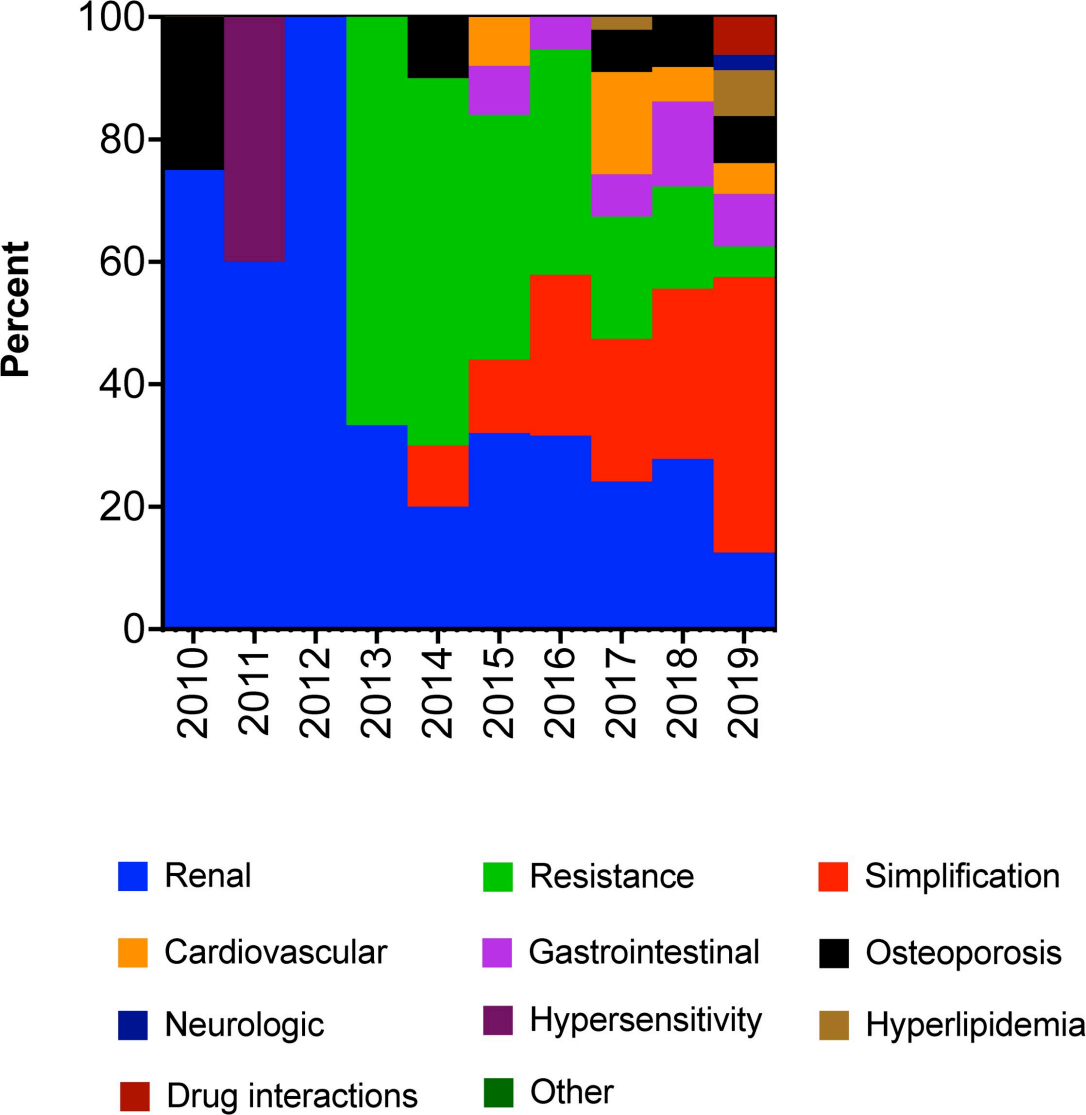
Reasons for 2DR initiation by indication. Each indication is represented as the percent of patients that were switched due to this indication in each year from 2010–2019. Some patients had more than one indication for 2DR switch.

The most common reasons for switching to 2DR were renal toxicity (26.1%), therapy simplification (25.0%) and resistance to prior ART (22.7%). Other, less common reasons were cardiovascular toxicity, osteoporosis, and gastrointestinal adverse effects. Forty patients featured more than one reason for 2DR initiation, all of which were documented (Table 2). The indication for changing patients to dual therapy changed over time. While resistance constituted a major indication in 2013–2015, treatment simplification became a major indication from 2018 (Fig 2). The combination of DTG+DRV was the most common regimen used in the ART resistant indication, while DTG+RPV treatment was the most common 2DR combination used in most other indications (Table 2).

**Table 2:** 2DR switches-reasons, regimens and patient characteristics.

Reason for 2DR initiation N (%)	Most common ART before switching to 2DR N (%)	Most common 2DR regimen N (%)	Mean Age Y (SD)	Gender M/F	Treatment before 2DR Y (SD)	Switches N (SD)	% Detec VL	Documented resistance %
**Renal 46 (26.1)**	2 NRTI + INSTI 23 (50.0)	DTG+RPV 26 (56.5)	51.5 (10.6)	37/9	6.6 (5.2)	1.6 (1.3)	15.6	15.2
**Simplification 44 (25.0)**	2 NRTI + INSTI 24 (55.8)	DTG+RPV 33 (76.7)	49.3 (14.1)	28/16	9.8 (6.3)	2.5 (2.2)	18.6	18.2
**Resistance 40 (22.7)**	2NRTI + INSTI 14 (35.0)	DTG+DRV 20 (50.0)	46.8 (11.4)	27/13	9.9 (6.1)	2.6 (2.4)	52.6	67.5
**Gastrointestinal 15 (8.5)**	2NRTI + INSTI 10 (66.7)	DTG+RPV 11 (73.7)	48	9/6	5.5	2.3 (3.2)	6.7	13.3
**Cardiovascular 13 (7.4)**	2NRTI + INSTI 7 (53.8)	DTG+RPV 10 (71.4)	57 (9.5)	11/2	9.8 (7.9)	2.27 (2.3)	25	15.4
**Osteoporosis 13 (7.4)**	2NRTI + NNRTI/INSTI 10 (66.7)	DTG+RPV 8 (61.5)	54.9 (10.9)	8/5	10.9 (5.37)	2.8 (2.3)	15.4	15.4
**Hyperlipidemia 8 (4.5)**	2NRTI + INSTI 3 (37.5)	DTG+RPV 4 (50)	48.8 (6.9)	7/1	8.8 (6.37)	1.6 (1.3)	0	12.5
**Neurological 6 (3.4)**	2NRTI + NNRTI 3 (42.8)	DTG+RPV 4 (57.1)	51.1	6/1	6.2	2.0(1.7)	50	0
**Hypersensitivity 5 (2.8)**	2NRTI + INSTI 2 (40)	RAL+DRV 2 (40)	48.6 (14.3)	4/1	6.7 (7.5)	2.2 (1.3)	80	20
**Drug interactions 4 (2.2)**	2NRTI + INSTI 2 (50)	DTG+RPV 3 (75)	48.75 (6.8)	2/2	13 (6.2)	3.67 (1.5)	0	0
**Unknown 24 (13.5)**	2NRTI + INSTI 10 (41.7)	DTG+RPV 12 (50)	50	21/3	8.6			

INSTI- integrase inhibitor, NNRTI- non nucleoside analog reverse transcriptase inhibitor, PI- protease inhibitor, NRTI- nucleoside analog reverse transcriptase inhibitor, N- number, SD- standard deviation, % detect VL- % of patients with detectable VL at 2DR switch.

Twenty-four patients (13.6%) experienced a second switch to a different 2DR, performed primarily for treatment simplification, hyperlipidemia, drug interactions, and renal toxicity. The majority of patients that underwent a second switch to 2DR (75%) were treated with a PI combined with INSTI as the initial 2DR and most of them were switched DTG+RPV as second 2DR. Nine patients (5.1%) were switched back to triple drug therapy, due to DDI, simplification, cardiotoxicity and other non-specified adverse events or unknown reasons (Table 3).

**Table 3:** Switch to second 2DR or 3DR.

N (%)	2^nd^ 2DR		3DR
	24		9
**1**^**st**^ **2DR N (%)**	PI+INSTI	18 (75)	2 (22.2)
	NNRTI+INSTI	(16.7)	4 (44.4)
	NRTI+PI	1 (4.2)	3 (33.3)
	2 PI	1 (4.2)	0
	NNRTI+PI	0	
**2**^**nd**^ **regimen N (%)**	PI+INSTI	11 (45.8)	0
	NNRTI+INSTI-	9 (37.5)	0
	INSTI+NRTI-	3 (12.5)	8 (88.8)
	NNRTI+PI	1 (4.1)	0
	PI monotherapy		1 (11.1)
**Reason for switch N (%)**	Simplification	9 (37.5)	3 (33.3)
	Hyperlipidemia	3 (12.5)	0
	Drug interaction	2 (8.3)	1 (11.1)
	Renal, GI, resistance, OP	1 each (4.2)	1 (11.1)
	CVD		1 (11.1)
	neurotoxicity		1 (11.1)
	AE- other		3 (33.3)

Switch to second 2DR or 3DR-regimens by class 1^st^ 2DR, regimens by class 2^nd^ 2DR, regimens of 3DR and reasons for switch. INSTI- integrase inhibitor, NNRTI- non nucleoside analog reverse transcriptase inhibitor, PI- protease inhibitor, NRTI- nucleoside analog reverse transcriptase inhibitor. N- number. GI- gastrointestinal, CVD- cardiovascular disease, OP- osteoporosis. Some patients had more than one reason for switch.

**Fig 3:**
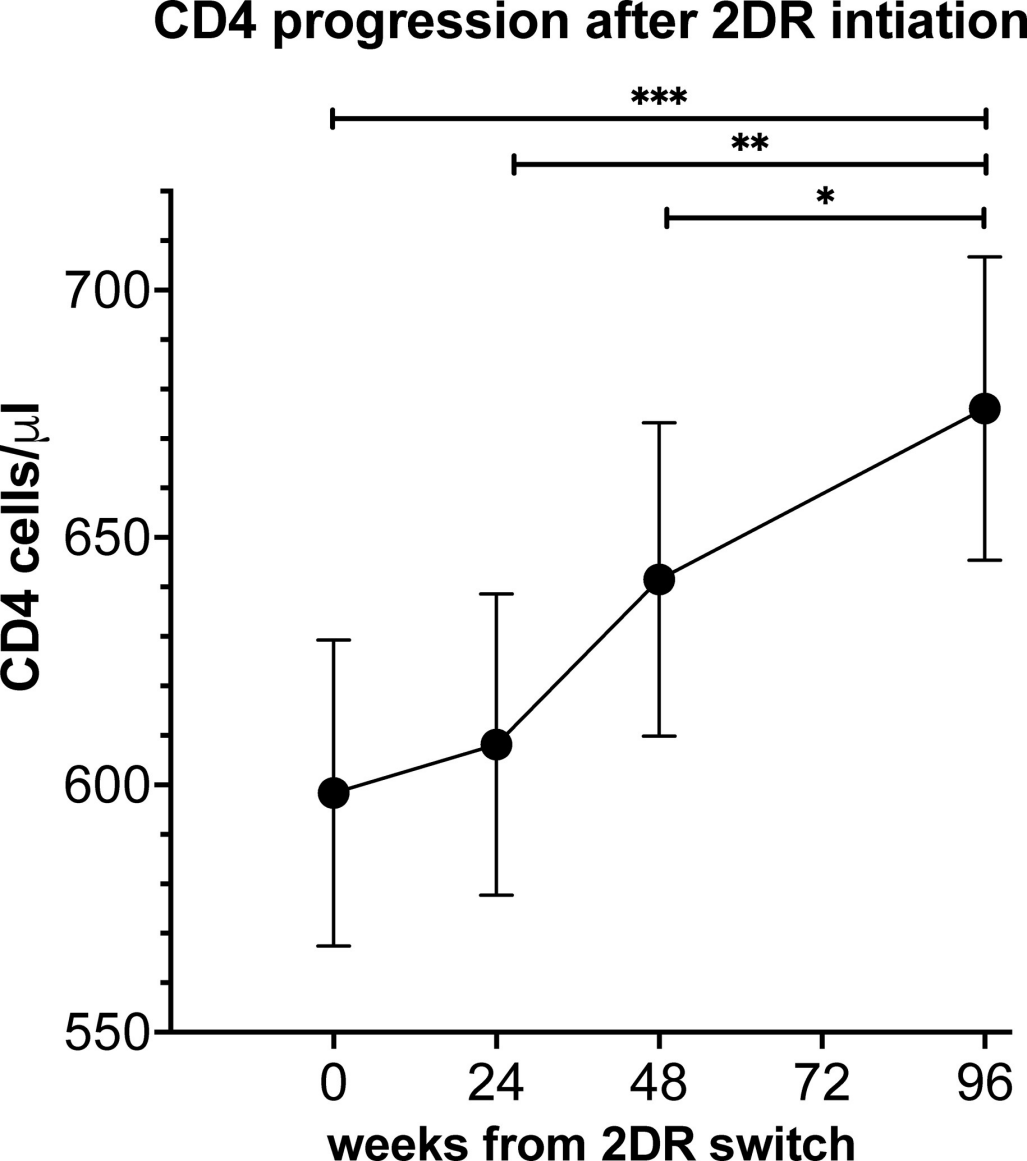
CD4 progression after switch to 2DR. 92 patients with consecutive CD4 measurements were included. Results are expressed ± SE. *** p<0.0001 ** p = 0.02 *p = 0.038.

Repeated CD4 count (cells/μl) measurements conducted in 92 patients with a complete follow up at 24, 48 and 96 weeks after 2DR initiation demonstrated a significant gradual increase in CD4 counts upon 2DR treatment from an average of 599 cells/μl at treatment initiation, to 614 cells/μl, 645 cells/μl and 680 cells/μl at 24, 48 and 96 weeks, respectively (p<0. 001, Fig 3). Seventy-two of these patients started 2DR with an undetectable VL, 17 were detectable and in 3 patients VL was not documented. Three naïve patients who started 2DR as their primary ART were excluded from this analysis. As corroboration of the above results, we performed a paired t-test in 140 patients that had follow ups of at least 96W but had some missing CD4 T cell values along this follow-up period, which indicated an increase in CD4 T cell counts from a mean of 583 cells/μl at initiation of 2DR to 654 cells/μl at 96 weeks (p<0.001).

To further assess the efficacy of dual therapy on viral suppression we binned VL values into three groups–“undetectable VL(UD)”, “low VL” (UD<VL<200), and “high VL” (VL = >200). Comparing the undetectable VL rate at 2DR initiation and 48 weeks demonstrated a significant increase in undetectability rate from 74.6% to 87.3% and a decrease in the low and high VL groups (P = 0.004). Significant results were also observed when comparing VL at 2DR initiation, 24 and 144 weeks of follow-up (86.1% & 87.0% P = 0.003 and p = 0.041, respectively).

**Fig 4:**
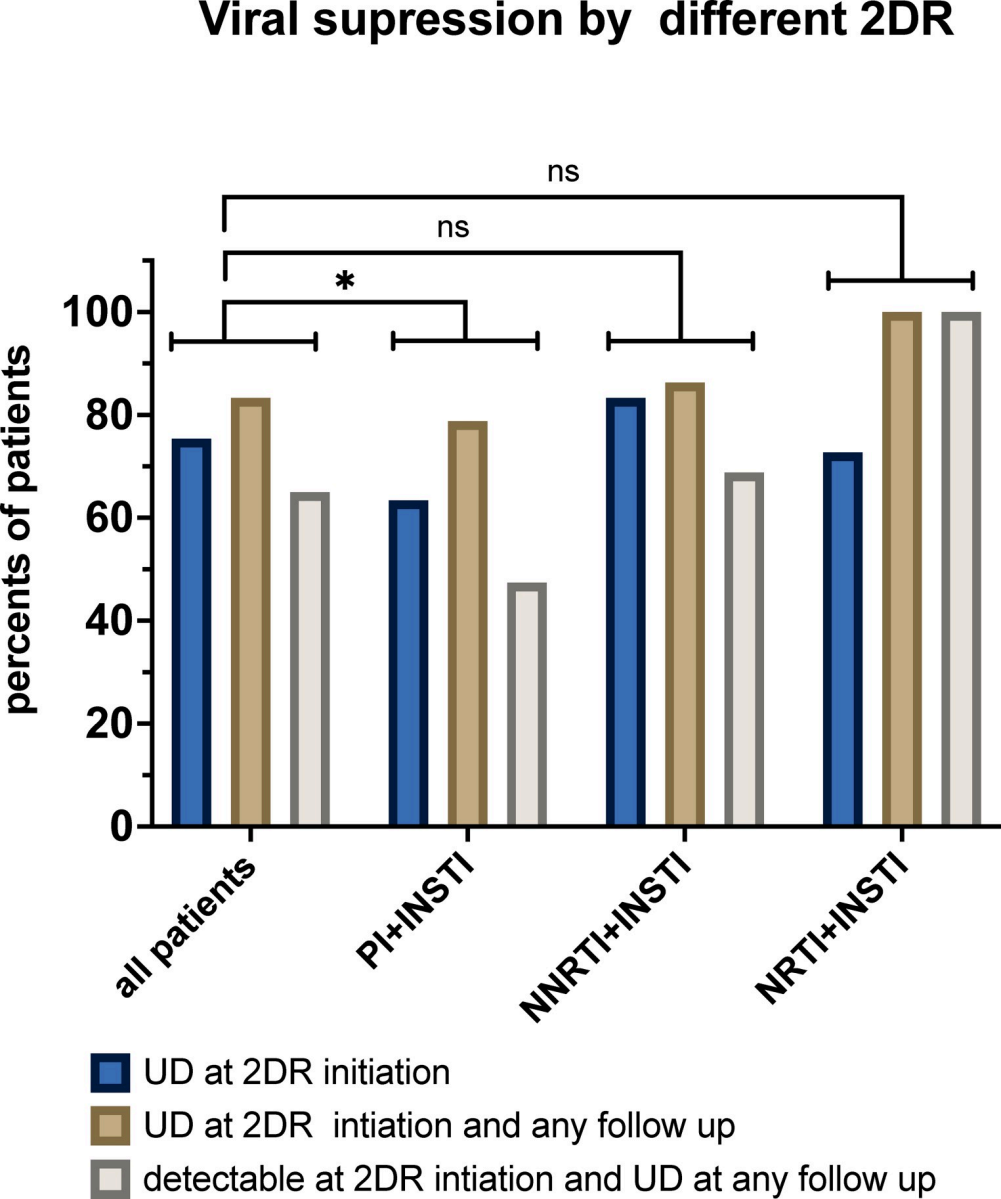
Viral suppression by different 2DR by class. UD at 2DR initiation- percent of total patients that started each 2DR combination. UD at 2DR initiation and at any follow up- percent of patients that started 2DR with undetectable viral load and were suppressed in every test- week 24, 48 96 and 144. Detectable at 2DR initiation and UD at any follow up- percent of patients that started with detectable viral load but later were suppressed in every test at week 24, 48 96 and 144. Patients that were changed to 2DR that was composed of PI and INSTI had lower rates of suppressed viral load and higher rates of failure in comparison to patients in other 2DR. p <0.05 McNemar test.

To differentiate the efficacy of different 2DR regimens, we compared the changes in viral suppression between patients who were switched to the newer and approved 2DR regimens dolutegravir+rilpivirine (DTG+RPV) or dolutegravir+lamivudine (DTG+3TC) and those treated by other 2DR regimens. As treatment simplification was the main indication to change to DTG+RPV or DTG+3TC, this patient subset featured a higher rate of undetectable VL at 2DR initiation (82.3% vs 62.6%, p = 0.006). We further compared the rate of patients who were undetectable at the 2DR initiation but became detectable at week 24, 48, 96 or 144 in each of the 2DR subsets. In the DTG+RPV and DTG+3TC group, the rate was significantly lower than in the other regimens (14.2% vs 28.5%, p = 0.007). Additionally, 72.2% of the DTG+RPV or DTG+3TC patients who initiated the 2DR with detectable viral load featured a completely suppressed viral load at all subsequent follow up time points, while the conversion rate to undetectability was only 52.2% in a group that included patients treated with other 2DR regimens. This difference did not reach statistically significance (p = 0.091), but the total effect indicated favorably to DTG+RPV and DTG+3TC treatment (p = 0.004). Application of the same analysis to other 2DR regimens indicated that 45% of patients treated with INSTI & PI, became completely suppressed, compared with an 73.9% in patients treated by other regimens (p = 0.038), (Fig 4). This analysis suggests that the of INSTI+PI combination is inferior in efficacy compared to other 2DR combinations, although the higher rate of failure could be attributed to the baseline higher prevalence of resistance (57% vs 13.8%) in this group of patients, and lower compliance (Table 4).

**Table 4:** 2DR regimens- patients characteristics and resistance profile.

	N	Age at 2DR Y (SD)	Years of HIV Y (SD)	Years of ART Y (SD)	N of switches (SD)	Reasons for 2DR	% Detect VL	% Resistance associated mutations				
								Any	NRTI	NNRTI	PI	INSTI
All patients	176	49.0 (12.1)	12.2 (7.7)	8.57 (6.2)	2.12 (2.1)	Renal 26% Simp 24.3%	24.2	27.2%	24.3%	14.7%	10.2%	1.7%
NNRTI+INSTI	102	49 (11.8)	11.8 (7.5)	8.86 (6.3)	2.12 (2)	Simp 34% Renal 27%	14.6%	13.6%	10.7%	5.8%	5.8%	1%
PI+ INSTI	54	47.4 (12)	12.5 (7.9)	8.6 (6)	2.2 (2.1)	Resis 50% Renal 24%	35.2%	57.4%	53.7%	35.2%	20.4%	3.7%
NRTI+ INSTI	11	60 (12.5)	12.7 (9.0)	6.5 (7.5)	1.67 (2.1)	Cardio 18% Renal 18%	30%	0%	0%	0%	0%	0%

2DR regimens- patients characteristics and resistance profile. INSTI- integrase inhibitor, NNRTI- non nucleoside analog reverse transcriptase inhibitor, PI- protease inhibitor, NRTI- nucleoside analog reverse transcriptase inhibitor. N- number SD- standard deviation, Reasons for 2DR- two most frequent reasons for switch in related group of patients, simp- simplification, resis- resistance, cardio- cardiovascular risk or disease, % detect VL- % of patients with detectable VL at 2DR switch.

Drug resistance associated mutations that were documented as naïve patients or due to virological failure, were documented in 48 (27.2%) of the patients and 24.3%, 14.7%, 10.2% and 2.3% of the patients had a least 1 mutation for NRTI, NNRTI, PI and INSTI respectively. The Mutation M184V in RT was documented in 18.6% of the patients and K103N in 4.5%. The rate of resistance was 40% in patients that were detectable at 2DR initiation with 32.5%, 22.2%, 7.5% and 5% of the patients harboring at least one mutation to NRTI, NNRTI, PI and INSTI respectively. As described above, resistance prevalence was 40% in patients that were detectable at 2DR initiation and 32% of the patients that were not completely virologically suppressed after 2DR switch had previously documented resistance- 27% for NRTI, 13.5% for NNRTI and 16.2% PI. No resistance mutations were documented to evolve in any of the patients that had detectable VL after 2DR.

One hundred and thirty eight patients had virological data at week 48, 18 of whom (13%) had a detectable viral load. Nine of these non-virologically suppressed patients, had a viral load of 0–200 copies/ml, while 9 featured a viral load of above 200 copies/ml. Eleven (61.1%) who featured a detectable viral load at week 48 of 2DR treatment also had a detectable viral load at treatment initiation. In comparison, only 25% of virally undetected patients at 48W featured a detectible viral load at treatment initiation (p<0.01). Surprisingly, the rate of documented resistance was lower (but non significantly) in the 48W detectable patients in comparison to the undetectable patients (16.6% vs 30.5% p = 0.414). Analysis of the patients who were detectable vs. undetectable in 96W yielded similar results. These results possibly stemming from adherence issues or resistance that was not documented due to low viremia.

A non-significant increase in triglycerides (p = 0.591), glucose (p = 0.562) creatinine (p = 0.426) and HDL levels (p = 0.355), and a decrease in AST (p = 0.41), ALT (p = 0.157) and total cholesterol levels (p = 0.834) were documented following the switch. Analyzing LDL levels in 46 patients with available results at 2DR initiation, 24W, 48W and 96W, of whom 23.9% were pretreated with PI, and in 26.1% PI was one of the 2DR components, indicated a significantly decreased from 112.98±30 mg/dl to 103.4± 29.8 mg/dl (p = 0.021). This effect also trended by comparing by paired T test of LDL at dual initiation and 96W of 79 patients (113.6±32 mg/dl vs 106.99±31.2 p = 0.067) but was not reproduced in 24W and 48W (p = 0.29 and P = 0.665 respectively).

## Discussion

Dual therapy for HIV-infected patients has been proven to be non-inferior to standard triple anti-retroviral therapy in randomized, double-blind controlled trials both in experienced [16] and naive HIV patients [9, 17, 19] while was unsuccessful in others [11, 20]. Currently, dual therapy (DTG+3TC) is recommended by many international guidelines for treatment initiation and other 2DR regimens are recommended as secondary treatment [21–23]. In Israel, 2DR was sporadically used in rare cases featuring significant drug resistance, even before 2DR was firmly established by large-scale trials [9], or made available in a convenient single tablet formulation (DTG+RPV or DTG+3TC) [17, 24]. The ensuing real-life adaptation of 2DR in several indications, as summarized in this country-wide survey, highlights some interesting and potentially important findings. First, efficacy of dual therapy in real life is supported both by significant CD4 increment after switching to 2DR, and by improved viral suppression in most regimens, but apparently lower efficacy in those combining INSTI with PI. Of note, in our study, INSTI combined with PI was provided to patients with higher rates of resistance mutations and detectable VL, which may reflect prior (and maybe current) low compliance, and potentially contribute to the different outcomes. Our results corroborate with other real life observational studies as the recent EuroSIDA report that indicated similar viral suppression rate and immunological response in 423 patients switching or starting 2DR, compared to 4327 naïve or experience patients who started 3DR regimens [25]. In our survey, the rate of 2DR usage was low in all HIV centers (1.6–4%), reflecting a more conservative approach and the lack of financial restrictions in choosing ART combinations in Israel.

Second, our study demonstrates that in many cases, the rationale for 2DR switch is motivated by a need to reduce previous treatment toxicity (58% of switches). In our cohort, we noted a trend towards 2DR improving hyperlipidemia, which reached statistical significance by 96W. This trend merits further larger-scale follow-up studies.

An observational study evaluating 121 patients treated with RAL combined with PI, reported that approximately 70% of them remained on this regimen with a median treatment duration of 60 months, with treatment simplification being the main reason for 2DR discontinuation in half the 2DR-failing patients [26]. Moreover, only three patients out of 81 (3.7%) who were switched to RAL/DRV/r experienced a virological failure [27]. Indeed, in our cohort, of 54 patients switching to a 2DR combining PI and INSTI, three (5.5%) featured a virological failure, and were switched back to 3DR, while 18 patients were switched to a second line 2DR, mainly due to a need for treatment simplification and 62.9% patients were maintained on PI+INSTI. Cappeti et al. reported similar results [13]. Likewise, Diaco et al. [28], reported only three patients returning to their previous 3DR and one patient experiencing viral failure (5.7%) in a single center 2DR de-escalation study. Although a major indication for 2DR was drug resistance in our cohort, analyzing undetectability at 2DR switch and during long term follow up, combined with similar rate of resistance, our results suggest that low adherence seems to be a major factor for virological failure that is observed in long term follow up 2DR,

Our study features several limitations stemming mainly from its retrospective design, including a lack of a control group treated with 3DR, and heterogenous ART regimens used before and after the switch to 2DR. Additionally, since 2DR was not prevalent before 2017, patients were excluded from our long-term metabolic and efficacy analysis due to a short follow up. Furthermore, due to the cross-sectional design of the study, we may have not identified patients that were switched to 2DR and switched back to 3DR before July 2019 and might have missed an excessive discontinuation rate of certain combinations.

In summary, our study indicates that two drug regimens are safe and effective in a real-life context, taking into account the impact of such treatment on virological suppression, immunological response and metabolic effects. The combination of PI and INSTI provided to a complex subset of patients was documented to be inferior to other 2DR combinations in this setting. Other long-term effects, such as enhanced cognitive impairment, the effect of viral transmission by sexual contact or from mother to child should be further explored by prospective, long term follow-up studies.

## Supporting information

S1 Dataset (XLSX)
